# Sub-microscopic *Plasmodium falciparum* infections and multiple drug resistant single nucleotide polymorphic alleles in pregnant women from southwestern Nigeria

**DOI:** 10.1186/s13104-024-06763-2

**Published:** 2024-05-09

**Authors:** Agatha N. Ibekpobaoku, Mary A. Oboh, Fatou Faal, Elizabeth Adeniji, Olusola Ajibaye, Emmanuel T. Idowu, Alfred Amambua-Ngwa

**Affiliations:** 1 University of Lagos, Akoka, Nigeria; 2 Medical Research Council The Gambia Unit (MRC), Banjul, Gambia; 3 Rochester Institute of Technology, Rochester, USA; 4 University of Medical Sciences, Ondo, Nigeria; 5 Nigerian Institute of Medical Research (NIMR), Yaba, Lagos Nigeria

**Keywords:** *P. falciparum*, Malaria in pregnancy, Malaria RDTs, *Pfdhfrs*, *Pfdhps*, Sulphadoxine Pyrimethamine, Single nucleotide polymorphism

## Abstract

**Objectives:**

The study evaluated sub-microscopic malaria infections in pregnancy using two malaria Rapid Diagnostic Tests (mRDTs), microscopy and RT-PCR and characterized *Plasmodium falciparum* dihydrofolate reductase (*Pf*dhfr) and *Plasmodium falciparum* dihydropteroate synthase (*Pf*dhps) drug resistant markers in positive samples.

**Methods:**

This was a cross sectional survey of 121 pregnant women. Participants were finger pricked, blood drops were collected for rapid diagnosis with *P. falciparum* histidine-rich protein 11 rapid diagnostic test kit and the ultra-sensitive Alere *Pf* malaria RDT, Blood smears for microscopy and dried blood spots on Whatman filter paper for molecular analysis were made. Real time PCR targeting the var acidic terminal sequence (varATS) gene of *P. falciparum* was carried out on a CFX 96 real time system thermocycler (BioRad) in discriminating malaria infections. For each run, laboratory strain of *P. falciparum* 3D7 and nuclease free water were used as positive and negative controls respectively. Additionally, High resolution melt analyses was employed for genotyping of the different drug resistance markers.

**Results:**

Out of one hundred and twenty-one pregnant women sampled, the SD Bioline™ Malaria Ag *P.f* HRP2-based malaria rapid diagnostic test (mRDT) detected eight (0.06%) cases, the ultra-sensitive Alere™ malaria Ag *P.f* rapid diagnostic test mRDT had similar outcome in the same samples as detected by the HRP2-based mRDT. Microscopy and RT-PCR confirmed four out of the eight infections detected by both rapid diagnostic tests as true positive and RT-PCR further detected three false negative samples by the two mRDTs providing a sub-microscopic malaria prevalence of 3.3%. Single nucleotide polymorphism in *Pf*dhps gene associated with sulphadoxine resistance revealed the presence of S613 mutant genotypes in three of the seven positive isolates and isolates with mixed wild/mutant genotype at codon A613S. Furthermore, four mixed genotypes at the A581G codon were also recorded while the other *Pf*dhps codons (A436G, A437G and K540E) showed the presence of wild type alleles. In the *Pf*dhfr gene, there were mutations in 28.6%, 28.6%, and 85.7% at the I51, R59 and N108 codons respectively. Mixed wild and mutant type genotypes were also observed in 28.6% each of the N51I, and C59R codons. For the *Pf*crt, two haplotypes CVMNK and CVIET were observed. The SVMNT was altogether absent. Triple mutant CVIET 1(14.3%) and triple mutant + wild genotype CVIET + CVMNK 1(14.3%) were observed. The *Pf*mdr1 haplotypes were single mutants **Y**YND 1(14.3%); N**F**ND 1(14.3%) and double mutants **YF**ND 4(57.1%); **Y**Y**D**D 1(14.3%).

**Supplementary Information:**

The online version contains supplementary material available at 10.1186/s13104-024-06763-2.

## Introduction

Nigeria contributes about 27% to the global malaria burden and 24% to malaria death globally [1]. Of the 33.2 million pregnant women in 2019, 35% (11.8 million) were exposed to malaria infection in 33 moderate to high transmission countries located in the World Health Organisation (WHO) Africa Region [1]. Pregnant women are among the high risk groups vulnerable to malaria infection due to their temporarily compromised immune system [2]. Pregnancy associated malaria (PAM) is a frequent occurrence in women living in malaria endemic countries especially in tropical and sub-tropical regions of the world such as Africa [3, 4]. PAM is defined as the detection of asexual parasite stages in peripheral or sequestered blood cells in the placenta of a pregnant woman [3]. *Plasmodium falciparum* is the most prevalent and implicated species in PAM and causes complications both in the mother and the foetus [5, 6]., sometimes leading to maternal anaemia, spontaneous abortion, stillbirths, premature and low birth weight [3, 6–10]. As a result of the presence of sub-microscopic infections in pregnant women, detection of malaria infections requires high sensitive diagnostic tools that can detect low parasite density [11]. Traditionally, microscopy is the gold standard, but due to the various challenges (inadequate training/experienced of microscopy readers, deficiency in personnels, sub-standard or inadequate equipment, lack of power supply etc.) that surrounds it, [12, 13] rapid diagnostic tool such as *P. falciparum* histidine rich protein II (HRP-2) malaria RDTs are deployed in many endemic areas where microscopy is unavailable. It requires less training, cost effective and produces timely result [14–16]. However, the circulation of parasite with deleted PfHRP2 gene makes parasite detection with mRDT difficult [17, 18] Therefore, more sensitive mRDT would be effective in providing timely results [19–20] WHO treatment policy for uncomplicated malaria in the general population is the use of artemisinin based combination therapy while in pregnant women, at least 2 doses of Intermittent Preventive Treatment with sulfadoxine-pyrimethamine (IPTp-SP) after quickening. In sub-Saharan Africa, IPTp-SP has been shown to reduce adverse infant and maternal outcomes such as maternal anaemia, low birth weight, placental malaria and perinatal mortality [21, 22], and neonatal mortality [23]. The implementation and effectiveness of this approach has been largely riddled by the development of *P. falciparum* resistance to SP [24, 25]. Single nucleotide polymorphisms in the *P. falciparum* dihydrofolate reductase (*Pfdhfr*) and the *P. falciparum* dihydropteroate synthase (*Pfdhps*) genes have been associated with resistance to pyrimethamine and sulfadoxine respectively. Substitutions at the *Pfdhfr* 51, 59, 108, 164, and *Pfdhps* 437, 540, 581 and 613 codons have been evaluated and detected in various populations in different endemic settings [26, 27]. Mutations at the *P. falciparum* multi-drug resistant gene 1 *Pfmdr1*) has been associated with various artemisinin partner drugs. For instance, substitutions in codons N86, 184 F and D1246 of *Pfmdr1* have been associated with resistance to lumefantrine and amodiaquine while the *Pfmdr1* 86Y allele has been strongly linked with chloroquine and amodiaquine resistance [44, 46]. Moreover, 1246Y alleles have also been shown to increase *P. falciparum* susceptibility to mefloquine, halofantrine and artemisinin [45]. While the resistance of *P. falciparum* kelch 13 propeller domain was uncommon, there were fixation in the prevalence of *Pfcrt* at codons 74–76 and 86Y and high prevalence of *Pfdhfr* and *Pfdhps* among Nigeria populations [47]. The NFD haplotype (86 N-184 F– 1246D) have been reported to significantly associate with treatment failure among children under five [26] in Nigeria. However, there is paucity of data of these resistant associated mutations in pregnant women from Lagos State, Nigeria. Therefore, this study was designed to i) evaluate sub-microscopic malaria infections in pregnant women using two mRDTs, microscopy and RT-PCR; ii). Characterize *Pfdfr*, *Pfdhps*, *Pfcrt* and *Pfmdr1* drug resistant markers in positive isolates.

## Materials and methods

### Study design, sites and participants

This was a cross-sectional study that involved pregnant women attending antenatal clinic in Epe and Ogudu Primary Health Centres, in Lagos State. The study sites are located in the south– western part of Nigeria and have high malaria transmission. The entomological inoculation rate as reported from a previous study [28] is 8.4 infective bites per person per month (ib/p/m) by human bait and 5.45 ib/p/m by pyrethrum sprays catch. The time frame for the collection of the pregnant women samples was from November, 2019 to March, 2021.

Written and verbal consents were obtained from all the participating pregnant women before the commencement of the study. Only consenting pregnant women with no pregnancy-related complications were enrolled into the study. Pregnant women not meeting study criteria were excluded.

After sensitisation and consenting, one hundred and twenty- one pregnant women of were recruited into the study.

### Blood collection

Participants were finger -pricked and blood drops collected for rapid diagnosis with *P. falciparum* histidine-rich protein II rapid diagnostic test kit and the ultra-sensitive Alere Pf malaria RDT, blood smears for microscopy and dried blood spots (DBS) on Whatman filter paper for molecular analysis were also collected. DBS were air-dried and kept in sealed plastic bags containing desiccants until use.

For pregnant women that tested positive, the attention of their attending obstetrician and gynecologist were brought to the situation and care thereafter were outside the purview of the study.

### DNA extraction and real time PCR (RT-PCR) for malaria molecular diagnosis

Genomic DNA of all samples was extracted from three punches of 3 mm dried blood spot using the QIAamp DNA Blood Mini Kit (Qiagen®, Hilden, Germany), eluted in a 100 µL final volume and stored at − 20 °C until use.

To accurately detect the presence of malaria parasites from these gDNA samples, RT-PCR targeting the *var* acidic terminal sequence (*va*rATS) gene of *P. falciparum* was carried out as detailed elsewhere [29, 30]. Briefly, 5 µL of template gDNA was added to a master mix containing 1 µL of nuclease-free water, 10 µL of 2x Taqman Universal PCR Mastermix (Applied Biosystems, New Jersey, USA), 1.6 µL of 10 µM forward and reverse primer each and 0.8 µL of 10 µM probe. The master mix together with the template gDNA was run on a CFX 96 real-time system thermocycler (BioRad). For each run, laboratory strain of *P. falciparum* 3D7 and nuclease free water was used as positive and negative controls respectively.

### High Resolution Melting (HRM) for *P. Falciparum* drug genotyping

Evaluation of single nucleotide polymorphism associated with resistance to pyrimethamine *Pfdhfr* (codons 51, 59, 108,164), sulphadoxine -*Pfdhps* (codons 436, 437, 540, 581, 613), artemisinin-partner drug such as lumefantrine and amodiaquine - *Pfmdr* (86, 184, 1042, 1246), and chloroquine *Pfcrt* (72–76) were done using the Qiagen TypeIT master mix. First, the primers were reconstituted and diluted to 10X, from this, 0.7µM and 1X of Qiagen master mix, 3.3 Μl of nuclease free water and 1 µL of gDNA (Supplementary Table 1) was used for the HRM drug assay as described earlier [31] For each drug target, mutant and wild type strains of laboratory cultured adapted parasites and nuclease free water were used as controls.

### Statistical analysis

Data was entered in excel and exported to Statistical Package for Social Sciences (SPSS) version 21.0 (SPSS, Inc. Chicago IL, USA) for analyses. The performance of each diagnostic tool was evaluated as per their sensitivity, specificity, positive predictive value (PPV), and negative predictive value (NPV) with RT- PCR as the gold standard. Agreement between pairs of evaluation tools was tested based on Cohen Kappa’s statistics where ≤ 0 indicates no agreement, 0.01–0.40 - slight to an average agreement, 0.41–0.80– moderate-stable agreement, and 0.81-1.00 perfect agreement [48].

Results of the drug resistant assay were scored using the Light-cycler software supplied with the machine after adjusting the sliding window to the appropriate melt curve. Samples with the same curve profiles with either the mutant or wild type controls were scored accordingly (wild or mutant or mixed in cases where it has the curves of both strain).

Further, amino acid mutation of the single nucleotide polymorphisms at each codon of each target molecular marker (*Pfcrt*, *Pfdhfr*, *Pfdhps* and *Pfmdr1*) were used in constructing the different haplotype per specific gene.

## Results

### Participants background information and parasite diagnosis

Majority of the pregnant women sampled was within the age brackets 30–34 (33.1%) and 25–29(33.1%). This was followed by the age brackets 20–24(21.5%) and 35–39(10.7%). Only one participant each falls within the age brackets 15–19 (0.8%) and 40–44 (0.8%). There was more Multigravida − 86(71.1%) than Primigravida– 35(28.9%) women in the study. In terms of pregnancy terms, the highest participants were on their second trimester (51.2%) followed by the third trimester (48.0%), while the least were the first trimester (0.8%). With regards to the use of IPTp with sulphadoxine pyremethamine, surprisingly, majority (59.5%) of them did not receive the preventive drug prior to the day that they were enrolled in the study, and only 24 (19.8%) of them had taken one dose, 18(14.9%) had taken two doses, while only 7(5.8%) of them had used three doses (Table 1 below).

**Table 1:** Clinical and Demographic Characteristics of pregnant women under study

Age	Kosofe	Epe	Total
15–19	0		1(0.8%)
20–24	9	17	26(21.5%)
25–29	23	17	40(33.1%)
30–34	14	26	40(33.1%)
35–39	6	7	13(10.7%)
40–44	0	1	1(0.8%)
**Total**	**52**	**69**	**121(100%)**
**Term of Pregnancy**			
First Trimester	0	1	1(0.8%)
Second Trimester	21	41	62(51.2%)
Third Trimester	31	27	58(48.0%)
**Total**	**52**	**69**	**121(100%)**
**Gravida**			
Primigravida	17	18	35(28.9%)
Multigravida	35	51	86(71.1%)
**Total**	**52**	**69**	**121(100%)**
**Number of IPTp + sp**			
None	6	66	72(59.5%)
One	24	0	24(19.8%)
Two	15	3	18(14.9%)
Three	7	0	7(5.8%)
**Total**	**52**	**69**	**121(100%)**

**Fig. 1:**
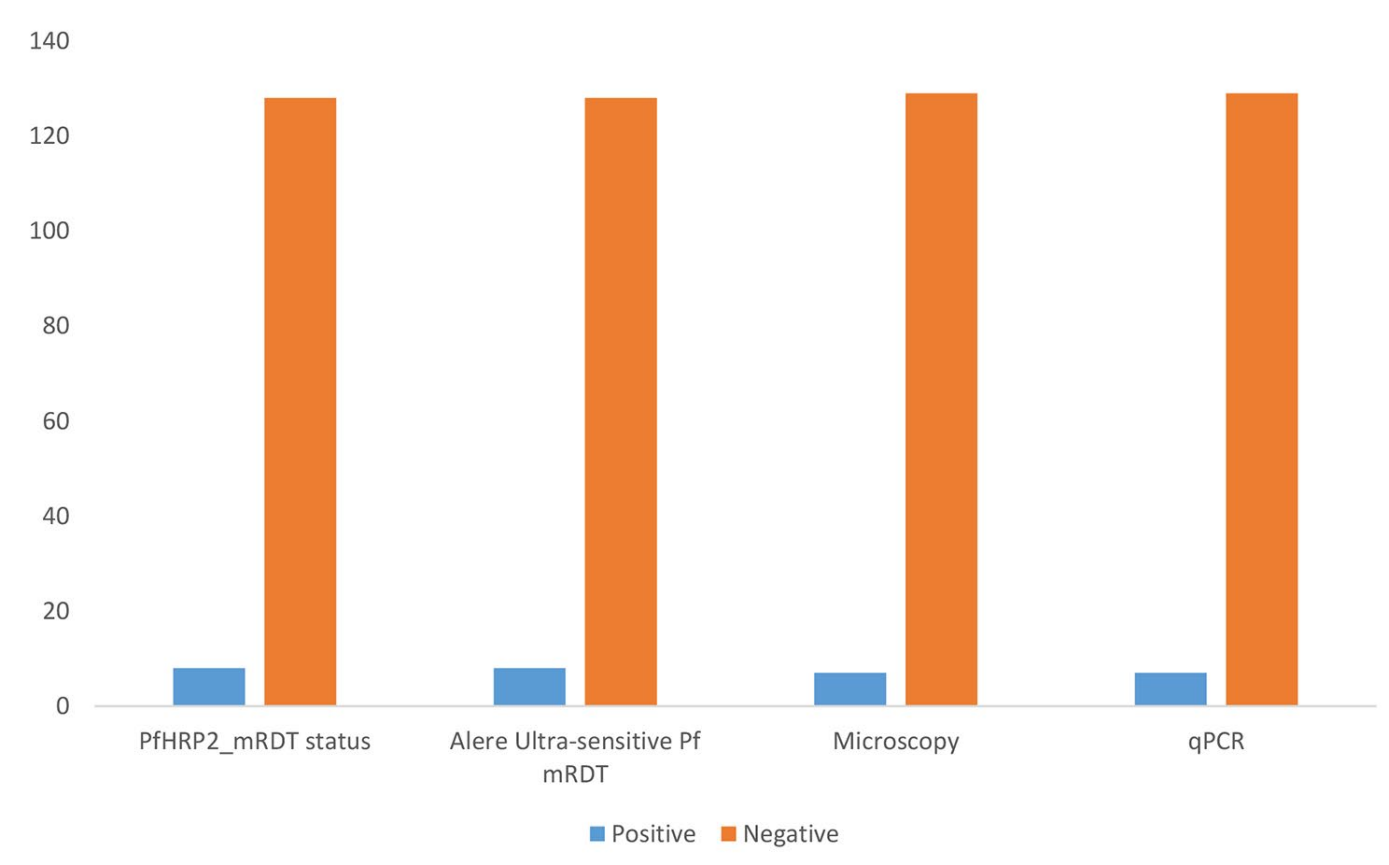
Showing the observed number of positives and negatives across the four diagnostic tools

One hundred and twenty-one (52 from Kosofe and 69 from Epe) pregnant women were recruited into this study. Of this, rapid diagnosis with *P. falciparum* HRP2-mRDT detected malaria infections in eight (0.06%) of them, the ultra-sensitive Alere™ Pf malaria RDT also gave similar outcome in the same samples as detected by the mRDT (Fig. 1). In contrast, microscopy and RT-PCR confirmed only four of the eight infections detected by both rapid diagnostic tests to be true positive and RT-PCR detected additional three samples not shown to be positive by either of the mRDT. Thus, employing RT-PCR as the comparator (gold standard), PfHRP2 and uRDT both showed high specificity (96.9% ), but their sensitivities were low (Table 2). However, the sensitivity and specificity of microscopy with regards to RT-PCR was almost perfect (96.69% and 96.58% respectively). For all further drug genotyping assay, only the seven samples positive by both microscopy and RT-PCR were included.

**Table 2:** Evaluation of the diagnostic performance of different malaria rapid diagnostic test kits and microscopy with real time PCR as the standard in P. falciparum infection from pregnant women

	Real-time PCR		Sensitivity (95% CI)	Specificity (95% CI)	PPV (95% CI)	NPV (95% CI)	Kappa	*P*-value
	Positive	Negative						
**PfHRP2_mRDT status**								
Positive	4	4	57.1 (18.4–90.1)	96.9 (92.3–99.2)	50 (23.9–76.1)	97.7 (94.7–99)	0.506	0.06
Negative		110						
**Alere Ultra-sensitive Pf mRDT**								
Positive	4	4	57.1 (18.4–90.1)	96.9 (92.3–99.2)	50 (23.9–76.1)	97.7 (94.7–99)	0.506	0.06
Negative	3	110						0.00
**Microscopy**	4	4	96.69 (39.76–100)`	96.58 (91.48–99.06)	66.67 (33.64–96.62)	100 (96.79–100)	0.65	0.13
	0	113						

### Drug resistant genotypes of *Pfdhps* and *Pfdhfr* from the study participants

Single nucleotide polymorphism in *Pfdhps* gene associated with sulphadoxine resistant revealed the presence of S_613_ mutant genotypes in three of the seven positive isolates and isolates with mixed wild/mutant genotype (A613S) at this codon. In addition, four mixed genotypes at the A581G codon was also recorded while the other *Pfdhps* codons showed the presence of wild type alleles.

**Fig. 2:**
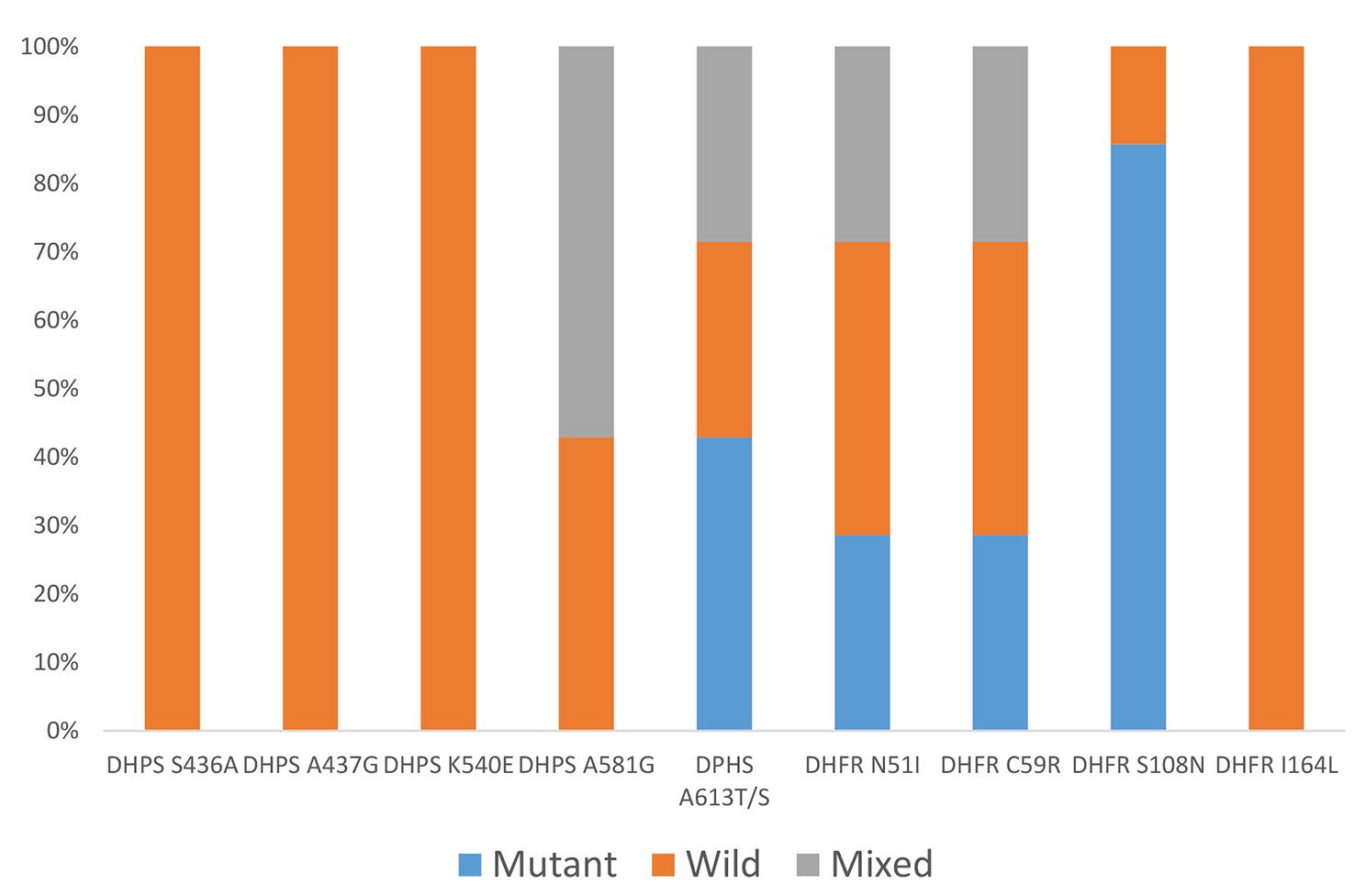
Bar chart of frequencies of wild, mutant and mixed alleleic infections in *Pfdhps* and *Pfdhfr* gene

In the *Pfdhfr* gene associated with pyrimethamine resistant, we observed mutations in 28.6%, 28.6%, 85.7% at the I_51_, R_59_ and N_108_ codons respectively. Mixed wild and mutant type genotypes were also observed in 28.6% at each of the N51I, and C59R codons respectively (Fig. 2).

### Profiles of malaria resistant markers of *Pfmdr1* and *pfcrt* from the study participants

**Fig. 3:**
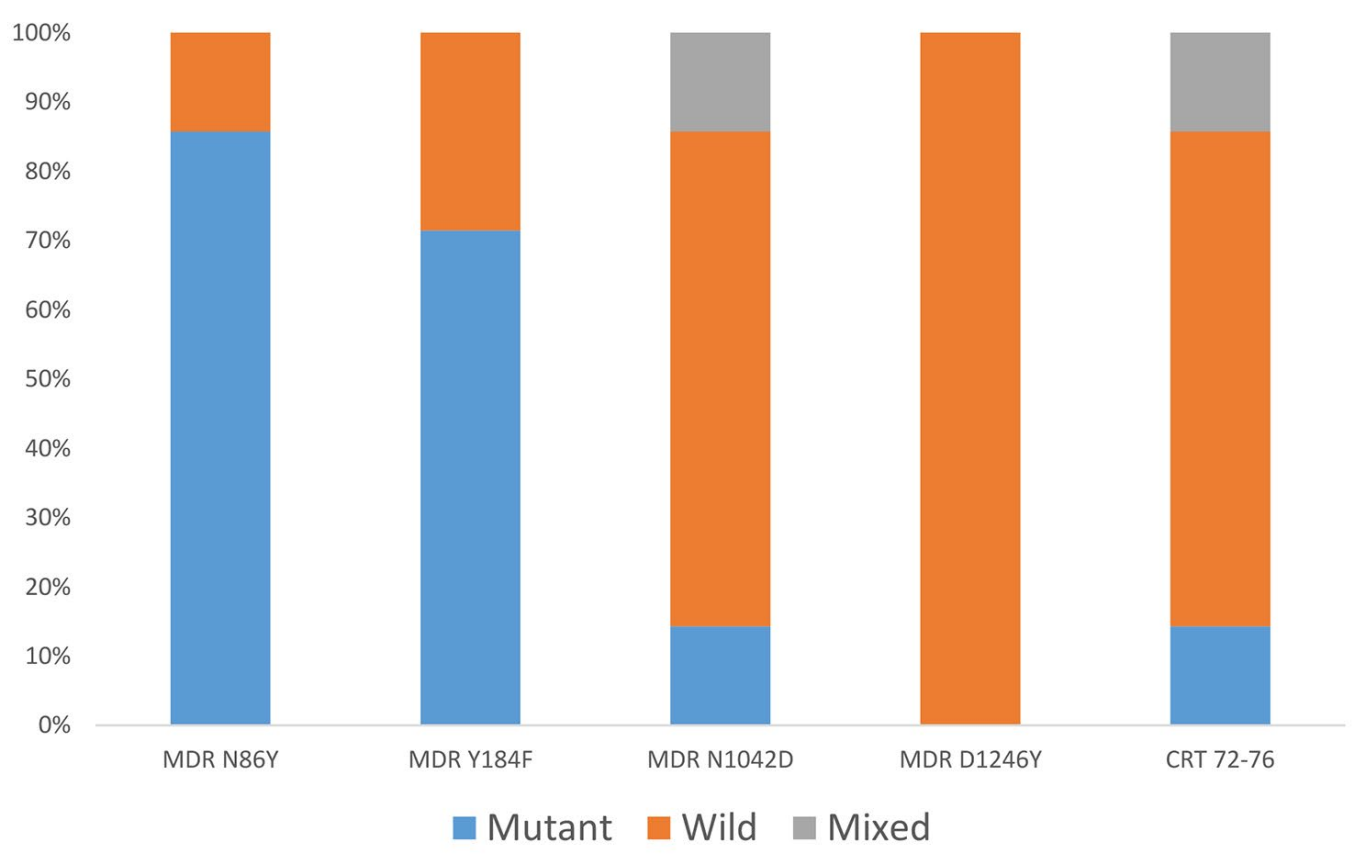
Bar chart showing frequency of wild, mutant and mixed allele infections in Pfmdr1 and Pfcrt

The *P. falciparum* multi-drug resistant gene 1 which has been associated with reduced parasite tolerance to amodiaquine and lumefantrine was also genotyped. Of the seven samples assayed, 85.7%, 71.4% and 14.3% harboured parasites that are resistant at the 86Y, 184 F and 1042D codons respectively. All parasites were of the wild type allele for the D1246 codon (Fig. 3). The *Pfcrt* mutant haplotype (CVIET) was observed in 14.3% of the isolates and a mixed mutant/wild genotype (14.3%) at these codons (72–76) was also observed (Table 3).

**Table 3:** Haplotypic distribution and prevalence of the different drug resistant genes from pregnant women in southwestern Nigeria

	Haplotype	Frequency n (%)
*Pfdhps*		
Single mutant	SAKA**S**	3 (42.9)
*Pfdhfr*		
Single mutant	NC**N**I	4(57.1)
Triple mutant	**IRN**L	2(28.6)
*Pfmdr1*		
Single mutant	**Y**YND	1(14.3)
Single mutant	N**F**N**D**	1(14.3)
Double mutant	**YF**ND	4(57.1)
Double mutant	**Y**Y**D**D	1(14.3)
*Pfcrt*		
Triple mutant	CV**IET**	1(14.3)
Triple mutant + wild genotype	CV**IET** + CVMNK	1(14.3)

### Haplotypic frequency and prevalence of the different drug resistant genes

Parasites with the *Pfdhps* single mutant SAKA**S** (with mutation at the 613 codon) were the most common occurring in 42.9% of the isolates while the single mutant *Pfdhfr* NC**N**I was the most common observed in these pregnant women, followed by the triple mutant **IRN**L (28.6%). In the *Pfmdr1* gene, double mutant (**YF**ND) was the most prevalent (57.1%) while the other single mutants **Y**YND, N**F**ND and **Y**Y**D**D occurred in similar proportion (14.3%). The CVIET triple codon mutation and the triple mutant + wild type has similar proportion (14.3%) (Table 3 above).

## Discussion

Malaria in pregnancy remains a public health challenge especially in malaria endemic areas such as Nigeria, and as a result of the growing evolution of malaria drug resistance, effective treatment of pregnant women with malaria is now more than ever threatened. The study evaluated four malaria diagnostic tools among pregnant women suspected of malaria infections and characterized *Pfdhfr, Pfdhps*, *Pfmdr1*, and *Pfcrt* drug resistant markers in malaria positive isolates. One hundred and twenty-one women were enrolled into this study and majority of them were in the age brackets 20–39 years. In addition, majority of the women had had multiple pregnancies before (multigravida), and unfortunately many of the recruited women had not taken IPTp-SP which poses a disturbing scenario, as this will result in the continuous transmission of *P. falciparum* with detrimental outcome to both the pregnant woman and the foetus. The study showed concordant results for both the *Pf*HRP2 and the ultra-sensitive Alere mRDTs in terms of sensitivity and specificity. This is similar to the findings of Unwin et al., 2020 [19]. Both of them showed high specificity and low sensitivity. In contrary, Briand et al., 2020 [32] reported that the ultra-sensitive rapid diagnostic test - uRDT specificity was slightly lower than that for conventional mRDT among Beninese pregnant women. The sensitivity from their study was high particularly among those in their first trimester, the multigravidae and asymptomatic. In addition, a Colombian study by Vasquez et al., 2018 also demonstrated a non-significant higher sensitivity of uRDT than Standard Bioline (sdRDT) [33]. Differences in transmission dynamics, endemicity and parasite density in these study areas could have been responsible for the variation in the performance of the mRDT tools.

Our microscopy and real-time PCR (RT-PCR) confirmed only four out of the eight infections detected by both mRDTs to be truly positive and further detected three samples also classified as negative to be positive for *falciparium* malaria. Misclassification of results as false negative by any of these mRDTs has serious implications to maternal and child health on one hand, and continuous malaria transmission on the other hand. The importance of diagnosis cannot be overemphasized as it is a prerequisite for treatment. Therefore, in order not to miss submicroscopic infections in pregnant women and avoid the deleterious effects of PAM, more sensitive tools should be employed. uRDT has been considered to be better in terms of sensitivity and specificity, however varying low sensitive and specificity results are presented from different studies and so there is need for re-evaluation of the efficiency of the uRDT. In addition to this it is very expensive when compared with the conventional mRDT.

Drug resistant typing was carried out on codons A613S, A581G, G436S, G437 and K540E for *Pfdhps* gene; N51,I C59R S108N, and I164L for *Pfdhfr* gene; 72–76 for *Pfcrt* and codons N86Y, Y184F, N1042D and D1246Y for *Pfmdr*1. Haplotypic distribution and prevalence of the different drug resistant genes were also estimated. Single nucleotide polymorphism (SNP) data from our study showed high prevalence of single mutant N108Y (57.1%), triple mutant N51I, C59R, S108N (28.6%) *Pfdhfr* alleles and single mutant A613S/T (42.9%) *Pfdhps* allele.

The single mutant haplotype- SAKA**S** in *Pfdhps* was the most prevalent haplotype (42.9%) while single mutation (NC**N**I) in *Pfdhfr* also was the most prevalent haplotype (57.1%) from our study. Our finding is dissimilar to that of Lucchi et al., 2015 [34] which noted that prevalence of these haplotypes in West Africa are generally high. Although, the prevalence observed here is low compared to a previous study conducted elsewhere in Nigeria; [26] in Equatorial Guinea [35] and in Democratic Republic of Congo [36]. This could be due to the small number of sample size used for the drug resistant assay in the present study. However, lower prevalence (26.5– 56.25%) has also been reported in other West African countries such as in Senegal [29] and in Bukina Faso [37]. In our study, wild type alleles were recorded for S436, A437 and K540 of *Pfdhps* and I164 of the *Pfdhfr.* Mixed wild and mutant alleles were recorded for A581G of the *Pfdhps* as well. As per the WHO recommendation [38]for the discontinuation of IPT-SP in areas where K540E mutation prevalence is > 95% and A581G > 10% constant surveillance should be taken seriously in the study areas to be able to identify and track any change in the distribution of mutation that would inform policy on the IPT-SP use.

In our study, polygenomic infection was observed in A581G, and A613S/T of the *Pfdhps* alleles and N51I, C59R and S108N of the *Pfdhfr* alleles. The mutations in these two (*Pfdhfr* and *Pfdhps*) alleles were high. This is in agreement with the findings of Adegbola et al., 2023 [47] which reported a relatively high prevalence of SNPs from both *Pfdhps* and *Pfdhfr* genes. They also reported a relatively high prevalence of SNPs from both *Pfdhps* and *Pfdhfr* genes. They also reported that sextuple and septuple contributed to about 25.0% of the *P. falciparum* isolates in their study and opined that their presence might pose a high probability of the malaria parasite becoming extensively resistant to SP in Nigeria.

Quadruple[ [26, 27] (reported earlier in Nigeria), quintuple and sextuple [31, 40, 41](reported in South and East Africa) mutant haplotypes which had been linked with both in vivo and in vitro SP resistance were not observed in our study [39–41]. 

For the *Pfmdr1*, out of the seven samples assayed, 85.7%, 71.4% and 14.3% harboured parasites with resistant alleles at the 86Y, 184 F and 1042D codons respectively. This is similar to the findings of Issa et al., 2022 and Adamu et al., 2020 [41, 42] where *Pfmdr1* 184 F and 86Y predominated in their studies in Niger Republic and Northern part of Nigeria respectively. The high prevalence of *Pfmdr1* 86Y alleles has been associated with chloroquine resistance. This might be an indication of the risk in the efficacy of Arthemeter Lumefanterine (AL). Prevalence of *Pfmdr1* N86 allele seen in our study might be suggestive of possible AL pressure in the population. The *Pfmdr1* double mutant 86Y/184F was found in 57.1% of our study. This is in line with the findings of Issa et al., 2022 and Tuedom et al., 2021 [42, 43]. While the S1034C and D1246Y mutations were detected in our study, they were not found in the study by Issa et al., 2022 [42]. For the *Pfcrt*, it has been established that the CVIET haplotype is widely prevalent in Nigeria from various studies [44–46]. Our study reported 14.3% CVIET haplotype distribution which is lower than what Issa et al., 2022 reported [42]. They reported a higher 33.07% isolates from their study that harboured the CVIET mutant haplotype.

## Conclusion

Although, this study is limited in the number of sample size included in the study and the geographical spread of the samples, however, it emphasize the importance of the use of high sensitive tools for the diagnosis of malaria especially because of the submicroscopic infections that might not be detected with less sensitive tools. Relying solely on the outcome of RDT should be done with caution since misclassification of results as false negative by the mRDTs is possible as evident in this study. This has implication to the maternal, neonatal and child health and ultimately will perpetuate the continuous transmission of malaria. The presence of polygenomic infection which is indicative of high parasite recombination events and the possibility of spread of mutant parasite strains due to high transmission has the potential of jeopardizing the use of SP- IPT_P_ in the study area. Therefore, continuous monitoring should be done so as to identify presence of mutation and take appropriate and prompt action especially considering the fact that SP is the only available preventive treatment for the pregnant women.

### Electronic supplementary material

Below is the link to the electronic supplementary material.

Supplementary Material 1

## Data Availability

The datasets generated and analyzed in this study have been included in this manuscript.

## Declarations

### Limitations of study

This study is limited in the number of sample size included in the study and the geographical spread of the samples because it was self-funded.

## Abbreviations

DNA: Deoxyribonucleic Acid
HRP-2: Histidine - Rich Protein 2
IPTp-SP: Intermittent Preventive Treatment in Pregnancy with Sulphadoxine Pyrimethamine
mRDT: Malaria Rapid Diagnostic Test
NPV: Negative Predicative Value
LSH&TM: London School of Hygiene & Tropical Medicine
MRC: Medical Research Council
NIMR: Nigerian Institute of Medical Research
PAM: Pregnancy Associated Malaria
*Pf*crt: *Plasmodium falciparum* Chloroquine Resistant Transporter
*Pf*dhfr: *Plasmodium falciparum* dihydrofolate reductase
*Pf*dhps: *Plasmodium falciparum* dihydropteroate synthase
*Pf*mdr1: *Plasmodium falciparum* Multidrug Resistance
PPV: Positive Predicative Value
RT-PCR: Real Time Polymerase Chain Reaction
uRDT: Ultra Sensitive Rapid Diagnostic Test
varATS: var Acid Terminal Segment
WHO: World Health Organization
