# Supplementary material for: Sub-microscopic Plasmodium falciparum infections and multiple drug resistant single nucleotide polymorphic alleles in pregnant women from southwestern Nigeria
Source: BMC Res Notes. 2024 May 9;17:129. doi: 10.1186/s13104-024-06763-2 (PMC11083805; doi:10.1186/s13104-024-06763-2)
Supplement: Supplementary file 1 — Supplementary Material 1 [file 13104_2024_6763_MOESM1_ESM.docx]

**Supplementary table 1a: 10X primer probe mix**

| ***Reagent*** | ***Stock concentration*** | ***Final concentration*** | ***Volume (μL)*** |
| --- | --- | --- | --- |
| PCR grade water or TE buffer | NA | NA | 60 |
| Forward Primer | 50 μM | 10 μM | 20 μL |
| Reverse Primer | 50 μM | 10 μM | 20 μL |
| **Total volume** |  |  | **100 μL** |

**Supplementary table 1b: Master mix preparation**

|  | **Final conc.** | **1X** | **100X** |
| --- | --- | --- | --- |
| 2X MM | 1X | 5 | 500 |
| 10uM primer (Q) | 0.7uM | 0.7 | 70 |
| H_2_O | - | 3.3 | 330 |
| DNA | - | 1 | - |
|  |  | 10ul |  |
